# The effects of domestication and ontogeny on cognition in dogs and wolves

**DOI:** 10.1038/s41598-017-12055-6

**Published:** 2017-09-15

**Authors:** Michelle Lampe, Juliane Bräuer, Juliane Kaminski, Zsófia Virányi

**Affiliations:** 1 Wolf Science Center, Dörfles 48, 2115 Ernstbrunn, Austria; 2 Radboud University, Department of Animal Ecology and Physiology, PO Box 9010, 6500 GL Nijmegen, The Netherlands; 3 Max Planck Institute for the Science of Human History, Department of Linguistic and Cultural Evolution, Kahlaische Strasse 10, 07745 Jena, Germany; 4 Institute of Psychology, Leutragraben 1, 07743 Jena, Germany; 5 Centre for Comparative and Evolutionary Psychology, University of Portsmouth, King Henry 1st Road, Portsmouth, PO12DY UK; 6 Comparative Cognition, Messerli Research Institute, University of Veterinary Medicine, Vienna, Medical University of Vienna, University of Vienna, Veterinärplatz 1, 1210 Vienna, Austria

## Abstract

Cognition is one of the most flexible tools enabling adaptation to environmental variation. Living close to humans is thought to influence social as well as physical cognition of animals throughout domestication and ontogeny. Here, we investigated to what extent physical cognition and two domains of social cognition of dogs have been affected by domestication and ontogeny. To address the effects of domestication, we compared captive wolves (n = 12) and dogs (n = 14) living in packs under the same conditions. To explore developmental effects, we compared these dogs to pet dogs (n = 12) living in human families. The animals were faced with a series of object-choice tasks, in which their response to communicative, behavioural and causal cues was tested. We observed that wolves outperformed dogs in their ability to follow causal cues, suggesting that domestication altered specific skills relating to this domain, whereas developmental effects had surprisingly no influence. All three groups performed similarly in the communicative and behavioural conditions, suggesting higher ontogenetic flexibility in the two social domains. These differences across cognitive domains need to be further investigated, by comparing domestic and non-domesticated animals living in varying conditions.

## Introduction

An organism’s behaviour and cognitive traits are shaped through selection pressures as well as through ontogenetic influences^1,2^. Animals are faced with social and physical challenges in their environment, ranging from finding food to cooperation among group members^3^. From both, evolutionary and ontogenetic perspectives, a more complex environment can advance social as well as physical cognitive abilities^4^. Domestication is a special case, as here, non-human animals adapt to the human environment. This adds new challenges and selection pressures that were not posed on the wild ancestors and may relax some of the requirements on traits that are critical for survival in the wild^5^. For instance, domesticated species may have acquired social skills to interact with their human partners (social cognition), and may have lost skills relating to independent problem-solving and understanding their physical environment (physical cognition).

Regarding social cognition, research in the last two decades shows that pet dogs are particularly sensitive to human communicative cues^6^. As such, they outperform other animals in following human gestures to objects^7–10^, and often use human-provided information only if they have been addressed through ostensive cues (calling their name, eye-contact, etc.) beforehand^11–13^. Dogs seem to develop these skills earlier than their closest wild-living relatives, wolves, even when the latter are extensively human-raised^14^. This indicates that selection pressures during domestication have influenced dogs’ ability to communicate with humans which, if enabled by human socialization, can be further improved by life-long experiences^7,15^.

Compared to dogs, wolves seem to benefit more from observing conspecific and human actions that are not directed at them. Wolves follow non-communicative human gaze more often than dogs, and seem to pay more attention when observing others’ behaviours, which they, in turn, use to skilfully solve tasks^16–20^. No research has specifically addressed, however, whether wolves can go beyond attending to others’ behaviour and infer the intention underlying this behaviour. Studies show that dogs do not differentiate between humans’ intentional and accidental actions^21–23^, but may interpret gaze as a cue of someone’s intention to approach a certain object^8,24^. Due to the limited data comparing wolves’ and dogs’ understanding of behavioural cues, it is currently unknown whether and how domestication has affected this domain of social cognition. However, given wolves’ higher attentiveness to conspecific and human behaviours in previous studies, there is reason to believe that wolves would outperform dogs in comprehending behavioural cues. Dogs on the other hand tend to ignore behavioural cues when the cue is not specifically addressed to them through eye contact^13^. In addition, it has been theorized that the developmental effects of living among humans could improve animals’ use of intentional behaviours, a phenomenon referred to as ‘enculturation’^25^.

Dogs perform rather poorly in tasks that require understanding causal connections or physical characteristics of objects^26^. Not even intensive training on object manipulation and solving physical problems improves their performance^27^. Further, pets living in close contact to humans learn to rely on human help instead of solving problems independently, more than pets living outside of the house^28^. This suggests that dogs that live more independently, could potentially be better problem-solvers. One evolutionary theory that explains dogs’ poor performance in the causal domain is the information processing hypothesis. This hypothesis suggests that selection pressures that advance causal understanding and thus problem-solving in wild animals, have relaxed on their domesticated counterparts due to a buffering effect of human care^29,30^. Moreover, dogs’ different feeding ecology as compared to wolves may have altered their performance in the causal domain^31^. As dogs scavenge for food in waste stably distributed around human settlements while wolves search for and hunt prey actively, the feeding ecology hypothesis proposes that dogs might have evolved reduced causal insight, persistence and exploration. Indeed, wolves were found to be more persistent and explorative than dogs when confronted with novel objects or environments^31,32^. Thus, a manipulative problem-solving task may not be the most useful method to compare physical cognition in dogs and wolves, as the better success of wolves may reflect their greater persistence in exploration, rather than a more advanced causal understanding.

Here we aimed to investigate how selection pressures during domestication and/or ontogenetic effects might have influenced dogs’ and wolves’ social and physical cognition. Within the social domain, we differentiated between the use of communicative and behavioural cues given by a human. Regardless of cognitive domain, animals needed to choose between two containers (one baited with food while the other was empty) cued differently in an object-choice task but did not need to manipulate an object to solve a problem. After the cue was performed, the animals could indicate their choice by touching one of the two targets fixed on the ends of a table, on which the containers were presented. This table was placed against a fence on the other side of which the animals were free to move.

To test for differences in social cognition, we differentiated between the use of communicative and behavioural cues given by a human who sat visibly behind the table. For the communicative cues, the experimenter repeatedly called the animal’s attention in order to cue it the correct choice (i.e. looking or pointing at the correct container), while for the behavioural ones, the experimenter showed behaviours that could indicate her intention to access one of the containers or its contents (i.e. reaching out to or trying to open the correct object). In contrast, to test the animal’s physical cognition, causal cues were provided while the experimenter was hiding under the table (i.e. a container producing noise while shaken versus a container that made no sound, an inclined shape versus a flat shape). In addition, all animals were tested in a control condition to check whether they could find the baited container based on smell. All animals were tested in 2 sessions consisting of 14 trials. To investigate based on which cues the animals could infer where the food was hidden, we recorded the number of correct choices in each of the four conditions (communicative, behavioural, causal and control). Furthermore, to measure how attentive the animals were we coded the proportion of time the animals spent in front of the testing table (position) and spent gazing in its direction (orientation).

To test for the effects of domestication on the three cognitive domains, we compared dogs (n = 14) and wolves (n = 12) raised and kept under identical conditions. Finally, to address the effects of living in human homes, we compared dogs socialized with humans but living in captive packs to enculturated pet dogs living in human families (n = 12). We expected that, (i) pet dogs would outperform pack dogs and pack dogs would outperform wolves in following more difficult human communicative cues whereas (ii) wolves would benefit more from observing behavioural cues and (iii) would be more successful in using causal cues than pack dogs, who would be more successful than pet dogs.

## Results

### Number of correct choices

#### Comparing the two cue types within each condition

In all cases, individual cues within their respective conditions were similarly scored on by pet dogs (GLMM: communicative cues: x^2^ = 0.068, p = 0.795; behavioural cues: x^2^ = 0.251, p = 0.617; causal cues: x^2^ = 0.998, p = 0.318), pack dogs (GLMM: communicative cues: x^2^ = 0.234, p = 0.629; behavioural cues: x^2^ = 0.056, p = 0.814; causal cues: x^2^ = 0.00, p = 1.00) and wolves (GLMM: communicative cues: x^2^ = 0.910, p = 0.340; behavioural cues: x^2^ = 0.182, p = 0.670; causal cues: x^2^ = 1.283, p = 0.257), justifying grouping them into the four cue conditions (see Supplementary Figs S3–S5).

#### Comparing groups and conditions

To investigate the effects of domestication, we made a pairwise comparison between pack dogs and wolves living under identical conditions, whereas to test for developmental effects we compared pet dogs and pack dogs. In general, wolves performed better than pack dogs (GLMM: z = 2.127, p = 0.033) but no interaction was found between groups and conditions. However, the four cue conditions differed significantly from each other (GLMM: x^2^ = 21.077, p < 0.001). To test our hypotheses on the groups’ performance for each cognitive domain, we compared the three groups in each condition separately.

#### Communicative cues

**Figure 1:**
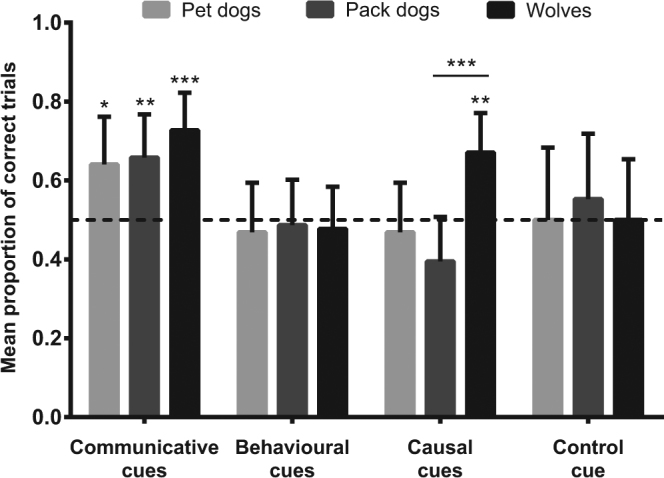
Mean proportion of correct trials (including 95% confidence interval) of every group in the four cue conditions. The dashed line indicates the chance performance level, with performances above chance shown by asterisk above the confidence bar, whereas differences between groups are indicated by a line with asterisk positioned above the two groups (*p < 0.05, **p < 0.01, ***p < 0.001).

No difference was found between the performance of wolves, pack dogs and pet dogs in the communicative condition (GLMM: x^2^ = 1.523, p = 0.47; Fig. 1). All three groups chose the indicated food location above chance (one sample t-tests: pet dogs: t_9_ = 2.882, p = 0.018; pack dogs: t_11_ = 3.742, p = 0.003; wolves: t_10_ = 4.822, p = 0.001).

#### Behavioural cues

All three groups performed similarly during the behavioural cues (GLMM: x^2^ = 0.046, p = 0.98; Fig. 1): none of them scored above chance (one sample t-tests: pet dogs: t_9_ = −0.580, p = 0.58; pack dogs: t_11_ = −0.352, p = 0.73; wolves: t_10_ = −0.690, p = 0.51).

#### Causal cues

For the causal cues, we found that wolves significantly outperformed pack dogs (GLMM: z = 3.486, p < 0.001; Fig. 1). The scores for the pack dogs and pet dogs were similar (GLMM: z = 0.881, p = 0.38). Only the wolves’ performance differed from chance (one sample t-test: pet dogs: t_9_ = −1.137, p = 0.29; pack dogs: t_11_ = −1.750, p = 0.11; wolves: t_10_ = 3.155, p = 0.01).

#### Control cue

There was no difference between the three groups in the control condition (GLMM: x^2^ = 0.1931, p = 0.91; Fig. 1), and none of them scored above chance (one sample t-tests: pet dogs: t_9_ = 0.00, p = 1.00; pack dogs: t_11_: 0.233, p = 0.82; wolves: t_10_ = 0.000, p = 1.00).

### Position

Pack dogs were positioned around the testing table relatively longer than wolves (LM: t = −4.305, p < 0.001). We found a significant effect for condition (LM: F = 13.036, p < 0.001) and an interaction between group and condition (LM: F = 2.158, p = 0.045) (see Supplementary Fig. S6).

### Orientation

**Figure 2:**
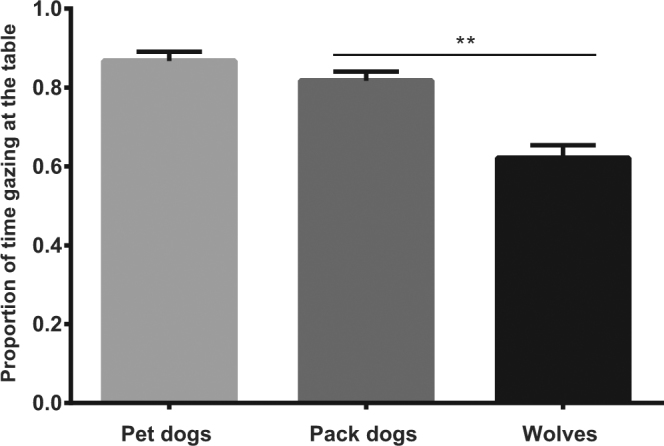
Mean proportion of time (including 95% confidence interval) that the groups gazed at the testing table. Significant difference is indicated by asterisk (*p < 0.05, **p < 0.01, ***p < 0.001).

Confirming the results on position, wolves spent less time watching the testing table overall than the pack dogs (LM: t = −3.353, p = 0.002; Fig. 2). Condition had a significant effect on orientation (LM: F = 22.281, p < 0.001), but there was no interaction between group and condition (LM: F = 1.832, p = 0.090).

For all statistical values linked to this data, see Supplementary Tables S3–S8.

## Discussion

We found that dogs and wolves were successful in using the experimenter’s communicative cues but did not follow her behavioural ones. In contrast only wolves succeeded in the causal condition, although they were less attentive during the cueing process (they stood less often in front of the testing table and gazed at it for a shorter time) compared to the dogs. In addition, the different experiences of pet and pack dogs did not influence their cognitive skills in this task, as the two groups performed similarly in all conditions. One may argue that due to a limitation on the selection of subjects, differences in composition of breed, sex and age between the two dog groups masked a possible effect of their different living conditions. However, former cognitive studies testing larger samples found no sex, no age and either no or only a weak breed effect in pet dogs^33–36^. Nevertheless, future research should aim at having more comparable groups of individuals, as the sum of these differences in characteristics may have impacted the overall performance on the group level.

Our results seem to imply that domestication impaired dogs’ ability to understand causal relationships, as in this condition, wolves outperformed pack dogs and were the only group to perform above chance level. One can argue, however, that the dogs were affected more by the sudden disappearance of the experimenter behind the table than the wolves. This may have caused the dogs to either give up, or to search for the human instead of paying attention to the cues. However, this latter hypothesis cannot explain former findings, where dogs failed to solve a puzzle box test even in the presence of an encouraging human, in contrast to wolves that performed equally well in human presence and absence^32^. Furthermore, if social dependence explained the poor causal performance of dogs^28^, the limited human contact of pack dogs and their freedom to manipulate objects in their large, natural enclosures independently from human presence and encouragement in our study, would have predicted they outperform pet dogs living in houses. However, we found no difference between the two dog groups, suggesting that dogs failed in the causal task for reasons other than being distracted by the social components of the test.

Instead, we propose that our finding complies with results of the social dog, causal ape study, in which apes (two other non-domesticated species) were more skilful in utilizing causal cues compared to dogs^29^. This seems to support both, the information processing, and the feeding ecology hypotheses^30,31^. We cannot exclude, however, that our results rather reflect a difference in the persistence of dogs and wolves to explore objects than in their physical cognition. Even though the animals did not need to solve an instrumental problem by manipulating an object, throughout their lives, the wolves might have learnt more about physical characteristics of objects by exploring them more persistently.

We also found that our extensively human-socialized wolves are as skilled in following communicative cues as dogs. It is well-established that adult wolves can follow the pointing of a human standing between two food locations^37,38^. However, this is the first study to show that wolves can use human gaze to locate hidden food as well as ipsilateral pointing of a person behind the non-indicated container. Even so, this success of wolves does not exclude the possibility that dogs, during domestication, evolved a genetic predisposition to learn faster about human communication^14^. We argue instead, that the socialization and training regime of the WSC allow the animals to learn a lot about human communicative cues. This is supported by the fact that the pack dogs used communicative cues as skilfully as pet dogs, although they had spent less time and participated in less diverse activities with humans.

Further research will have to clarify whether dogs and wolves interpret these cues in a similar way and to what extent the raising and keeping conditions of the animals influence their performance as well as its underlying cognitive mechanisms. It is always a challenging question to what extent study populations are representative of a species^39^. Nevertheless, our data suggests that some abilities that enable the use of human gaze and gestures are present in both these domesticated and wild canids. This agrees with the canine cooperation hypothesis that argues that high social attentiveness, tolerance and cooperation in wolves contributed to their competence to learn about human behaviour and possibly provided a good basis for dog-human cooperation to evolve^40^.

Finally, all dogs and wolves failed to make the right choices in the behavioural condition, which contradicts our prediction. There are several possible explanations for these results. First, the cues may have been too human specific and showed behaviours that animals could not identify as such. However, in a study prior to this one, dogs proved to be successful in the reach cue, though the unbaited side was left unattended^29^. Here we could argue that the animals made a connection between the hand and the baited container, and followed the hand as an indicative marker (see also ref.^21^).

A second explanation could be that all three groups were human-socialized to the point that they would use a human-given cue only if it had been addressed to them. Our prediction was that wolves would make use of watching others’ intentional actions also in the absence of human ostensive cues^20^. We would need to conduct further research to point out which factors influence the use of behavioural cues in dogs and wolves.

In conclusion, our results confirm that wolves can adapt their social cognitive abilities to their social environment, in this case to humans and their communication. Possibly for this reason, we found no evidence that domestication has altered how dogs use human-given cues. On the other hand, we found that domestication has left a mark on how dogs perform in a causal task. To place this in a broader perspective, social cognition seems to be subjective to individual learning, upbringing and experiences in life, whereas we found no evidence of such developmental processes on dogs’ causal cognition. In an extension to the social dog, causal ape theory^29^, we can sum up our results in the social canine, causal wolf hypothesis. Here we propose that socialized canines in general, are sensitive to human communicative cues, and that the skills underlying this comprehension likely facilitated domestication. Moreover, we suggest that domestication has reduced the independent problem-solving abilities of dogs within the causal domain. Whether this has happened by relaxing selection on their causal understanding or on exploring objects, needs further research.

## Methods

### Ethical statement

Pet dogs were tested with the consent of their owners prior to the test. All animals had ad libitum access to water and were not food deprived at any time. No special permission for the use of animals in such socio-cognitive studies is required in Austria (Tierversuchsgesetz 2012 – TVG 2012). The Tierversuchskommission am Bundesministerium für Wissenschaft und Forschung (Austria) is the relevant committee that allows research to run without special permission regarding animals, thus, this research complies with the current Austrian laws on animal protection. All methods were carried out in accordance with relevant guidelines and regulations.

### Subjects

12 captive wolves (mean age = 4.9 years), 14 pack dogs (mean age = 3.2 years) and 12 pet dogs (mean age = 7.6 years) were tested individually in this study. All subjects had experiences with various behavioural and cognitive tests run weekly. For information on individual characteristics such as sex, breed, origin and relatedness to other tested individuals, see Supplementary Tables S1 and S2.

### Experimental set-up

**Figure 3:**
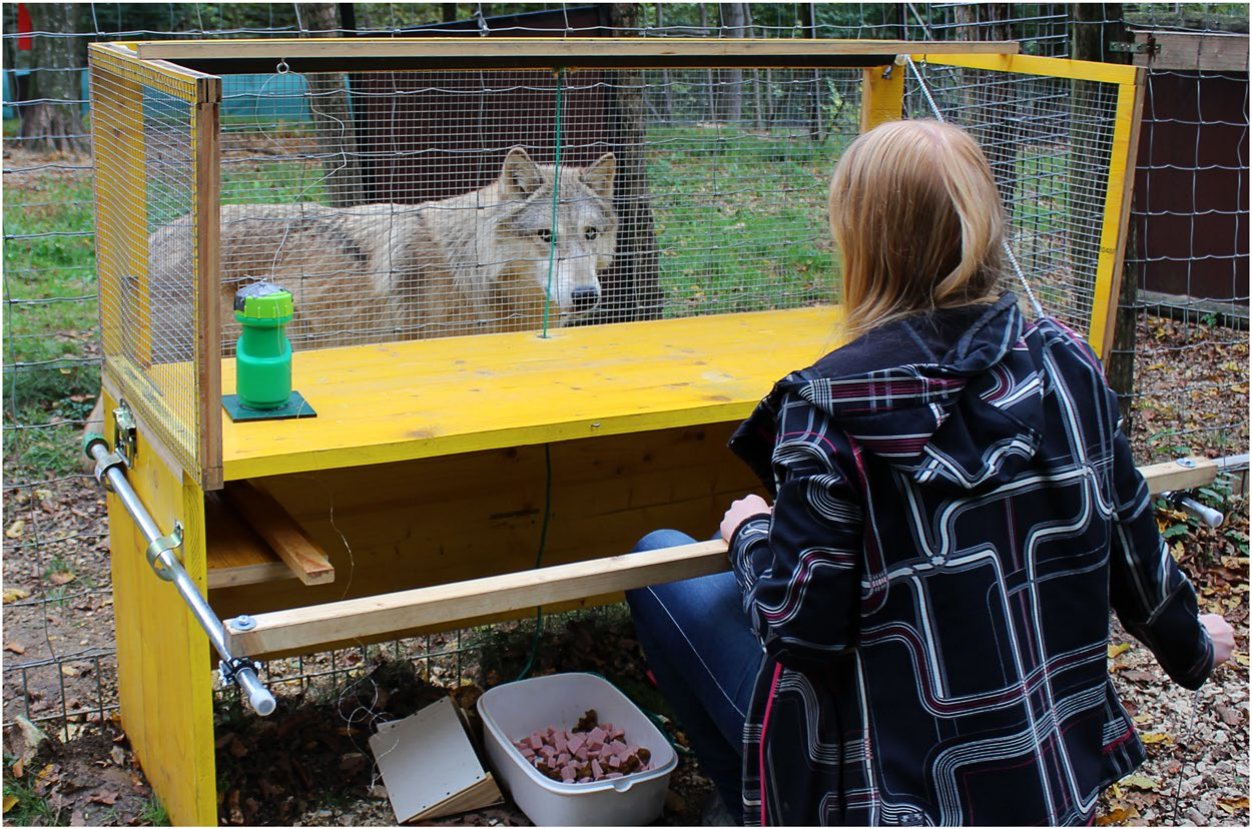
The experimental set-up of the test.

Subjects were tested in an outdoor fenced compartment (3 × 6 m) by one female experimenter (ML). Figure 3 shows the testing table (150 cm length × 50 cm width × 113 cm height) that was placed outside the wire mesh fence. The table was equipped with a screen of 146 × 57 cm, which could be pulled down by a cord to occlude the animals’ view of the table top.

Depending on the condition, two containers or concealer objects were placed at two ends of the table, with one of them containing food or blocking food from view, the other being empty. After a cue was given, subjects could indicate their choice by touching a target fixed on each side of the table or the apparatus itself close to the chosen object.

### Procedure

The animals were tested in an object-choice task in which they could choose between two objects placed on a table, after watching the experimenter giving either communicative, behavioural or causal cues. These cues indicated where food could be found. All animals were tested in all cue conditions, including a control condition with no information provided. Each animal was tested in 2 sessions, which consisted of 14 trials each.

### Pre-test

Animals were tested for motivation and attentiveness before every session. A piece of sausage was visibly put on the right or left side of the table. If the animal chose correctly, the experimenter rewarded the animal by giving it the piece of sausage through the table’s food opening. As soon as an animal succeeded four consecutive times, the experiment commenced. If the subject did not succeed four consecutive times within 15 trials, it was not tested that day.

### Experimental trials

Each trial started by lowering the screen to block the animal’s view of the table. Depending on the cue, the experimenter hid a piece of sausage in or behind one of the objects and placed them in predetermined positions. The experimenter drew the animal’s attention by shaking a bowl of food, before the screen was lifted and cueing started. During the communicative and behavioural conditions, the experimenter was visibly sitting behind the testing table, whereas she was hidden underneath it during the causal and control conditions. Each cue ended when the two targets on the sides of the table were pushed through the fence for choice-making. When the choice was correct, the animal was rewarded; when incorrect, the experimenter shut the screen and set up for the next trial. If no choice was made within 30 seconds, the trial was ended by pulling down the screen and the cue was repeated. When an animal was too distracted or disinterested to continue, the session was terminated and resumed on the next testing day.

Subjects were tested in 2 sessions, which consisted of two blocks; one for social (communicative and behavioural) and the other for asocial (causal and control) cues. The order of blocks within a session was counterbalanced, while the order of cues as well as the location of the hidden food within a block was predetermined and remained constant. Both sides of the table were enhanced for all cues (except for the look cue due to its minimal local enhancement), to rule out the possibility that animals only acted upon locally enhanced sides. This was accomplished by positioning the experimenter behind the empty container or by manipulating both containers.

All animals were tested in the following conditions (see Supplementary Video):

### Communicative cues

#### Look

The experimenter (E) was positioned behind the middle of the table with her hands down. The cue started as soon as eye contact was made with the subject. E nodded once with her head while gazing into the eyes of the subject for 1 second, after which she shifted her gaze to the cup containing food for 2 consecutive seconds. This process was repeated four times, after which E looked down.

#### Point

E was seated behind the empty cup, made eye contact with the subject after which she nodded once at the animal. She then pointed four times to the cup containing food with the index finger of her ipsilateral hand. Every pointing gesture was maintained for 3 seconds, before E pulled back her arm, nodded at the animal and pointed again. E kept gazing at the animal and only pointed when the animal was looking. After the cue, E placed her hand under the table and looked down into her lap.

### Behavioural cues

#### Reach

E sat behind the middle of the table, and briefly looked at the empty cup, then desperately stretched her arms to grab the baited cup for roughly 10 seconds. However, the cup was unreachable and E fell off the chair in the direction of the empty cup.

#### Open

E sat behind the middle of the table and leaned over to one side, picked up the empty cup, sniffed at it for roughly 10 seconds and placed it back disappointedly. Subsequently, she leaned over to the other end, picked up the baited cup, sniffed at it excitedly and tried to open the cup by turning its lid for about 10 seconds. After being unsuccessful, E put back the cup and looked down.

### Causal cues

#### Noise

E hid underneath the table and pulled a fishing line tightened over the frame above the table, thereby lifting the container attached by its lid. E shook the baited container with food and stones for roughly 10 seconds to create a noise. She then carefully released the string to place the baited container back on the table and repeated the procedure for the empty container.

#### Shape

Two wooden shapes were presented to the animal for approximately 5 seconds, one of which was an inclined shape that hid food, while the other was a flat piece of the same size.

### Control cue

#### Control

Two identical rectangular blocks of wood were presented to the animal for approximately 5 seconds, with one wooden block hiding a piece of sausage directly behind its middle to control for smell.

### Behavioural coding and inter-observer reliability

Videos were coded for the animal’s choice, position and orientation during each cue using Solomon Coder (http://solomoncoder.com). Choice was defined as the side first approached after the targets were presented. The relative duration (%) was coded for the duration that animals positioned themselves in front of and oriented themselves towards the testing table.

One person coded all videos, while a second independent observer scored randomly selected samples of 20% of all videos to assess inter-observer reliability. Reliability was excellent for choice, position and orientation (Cohen’s κ = 0.923, N = 210; κ = 0.833, N = 16510; κ = 0.790, N = 16510, respectively).

For additional information on the subjects, experimental set-up, procedure, experimental trials, behavioural coding and the data analysis, see the Supplementary Methods.

### Data availability

All data generated and analysed during this study are included in this published article (and its Supplementary Information files).

## Electronic supplementary material

Supplementary Video

Supplementary Materials

Supplementary Dataset 1
