# Supplementary material for: The effects of domestication and ontogeny on cognition in dogs and wolves
Source: Sci Rep. 2017 Sep 15;7:11690. doi: 10.1038/s41598-017-12055-6 (PMC5601471; doi:10.1038/s41598-017-12055-6)
Supplement: Supplementary file 2 — Supplementary Materials [file 41598_2017_12055_MOESM2_ESM.doc]

The effects of domestication and ontogeny on cognition in dogs and wolves

Michelle Lampe*^1,2^, Juliane Bräuer^3,4^, Juliane Kaminski^5^, Zsófia Virányi^1,6^

^1^Wolf Science Center, Dörfles 48, 2115 Ernstbrunn, Austria

^2^Radboud University, Department of Animal Ecology & Physiology, PO Box 9010, 6500 GL, Nijmegen, The Netherlands

^3^Max Planck Institute for the Science of Human History, Department of Linguistic and Cultural Evolution, Kahlaische Strasse 10, 07745 Jena, Germany

^4^Institute of Psychology, Leutragraben 1, 07743 Jena, Germany

^5^Centre for Comparative and Evolutionary Psychology, University of Portsmouth, King Henry 1^st^ Road, Portsmouth, PO12DY, UK

^6^Comparative Cognition, Messerli Research Institute, University of Veterinary Medicine, Vienna, Medical University of Vienna, University of Vienna, Veterinärplatz 1, 1210 Vienna, Austria

**Supplementary methods**

*Subjects*

All wolves (n = 12, timber wolves) and a subset of the pack dogs (n = 9, all mongrels born in Hungarian shelters) were separated from their mothers once they were 10 days old to be hand-raised under comparable conditions at the Wolf Science Center (WSC) in Ernstbrunn, Austria (for details see Range & Virányi 2013). The remaining pack dogs (n=5) were born at the WSC and in their first 5 months, were hand-raised during the day (from 9:00 till 17:00), but brought back to their mothers for the night.

All wolves and pack dogs live in stable packs, with sizes varying between 2 and 6 animals. Dog enclosures consist of 2000 to 4000 m^2^ large areas with natural vegetation and 2 to 3 shelters which are heated in winter, whereas wolf enclosures are 4000 to 8000 m^2^ in size with 1 to 2 unheated shelters. Water is permanently available in all enclosures. Dogs are fed once a day with a diet of primarily dry food, while the wolves are fed every 3 to 4 days with mainly carcasses of chickens, rabbits or deer. All WSC animals have been taught to follow commands like sit, down, paw and roll-over and are trained to do so daily. All dogs and wolves are also trained to walk on a leash. This training has allowed animals to be cooperative and attentive during tests, to do veterinary checks without sedatives and to interact with people for touristic purposes.

Pack dogs and wolves were separated from their pack members and participated in tests and training on a voluntary basis. Prior to testing, each subject was called into a shifting system that is present in each enclosure. Both pack dogs and wolves were then leashed by a familiar trainer and walked to the test enclosure where tests are usually held. This test enclosure is situated in a way that other animals from nearby enclosures cannot be seen or interacted with during the test. Pet dogs belonged to the personnel of the WSC and were familiar with the general environment and enclosures of the testing area. In contrast to the pack dogs, pet dogs lived with their human partners in a domestic environment. However, all pet dogs were used to being separated from their owners during tests, and thus did not show any stress signals due to social isolation. All animals had extensive experiences in cognitive research prior to this experiment.

Data were collected from the beginning of July till the middle of August 2015 (pet dogs), from the beginning till the end of September 2015 (pack dogs) and from the end of September till the beginning of November 2015 (wolves).

*The experimental set-up*

Subjects were tested in an outdoor, fenced compartment (3 x 6 m) by one female experimenter (ML). Exceptions were made for three wolves that had shown signs of stress when being enclosed in a small pen. Two wolves, Una and Wamblee, were tested with the shift to the larger neighbouring compartment opened, providing them with an additional area of 27 x 25 m, while the third wolf, Kenai, was tested in his own living enclosure of 8000 m^2^.

The testing table (150 cm length x 50 cm width x 113 cm height) was placed behind the wire mesh fence. The table was equipped with a screen of 146 x 57 cm, which could be pulled down by a cord to occlude the animals' view of the table top.

Two containers were attached to a long fishing line with their lids to enable manipulation. The fishing lines were tightened through eye bolts on a wooden frame on top of the apparatus, from where they reached the ground. In this way, the containers could be lifted from underneath the table. From this position, the screen could both be opened and shut by separate cords as well. After a cue was given, the experimenter pushed two targets through the mesh of the fence for the animal to indicate its choice. Both targets were attached to the ends of poles, which were held into place by two brackets on each side of the table. The poles were connected to each other by a target bar, which was used to move the two targets simultaneously. If an animal made the correct choice, it was rewarded with a piece of sausage (cube of 2 x 2 x 2 cm) which was placed in the hollowed end of a wooden reward bar under the testing table, which could be shifted through an opening (5 x 5 cm) directly under the container of choice. The reward bar moved through the mesh of the fence for animals to receive the sausage. Supplementary Figure S1 shows a detailed illustration of the testing table with all its assets.

All subjects were target trained since they were puppies; animals touched a target the trainer pointed to with the command “touch”. Thus, animals did not need to be trained to skilfully use the targets prior to the test. All animals were trained, however, until they were familiar with the apparatus, and in case had shown signs of being scared to approach the testing table, until they no longer showed fearful reactions toward it. Subjects were trained by professional animal trainers of the WSC who raised, trained and/or handled the pack dogs and wolves from an early age on, and thus, had a good relationship with the animals. During training, food was placed visibly on the table as during pretesting. During pretesting, only the downward and upward movements of the screen were new to the animals; few animals showed avoidance behaviours towards these, which was easily eliminated by rewarding the animals for staying close to the testing apparatus during screen movement.

Depending on the condition, two containers or concealer objects were placed at two ends of the table, with one of them containing food or blocking food from view, the other being empty. These objects were either plastic containers with lids (each 8 x 17 cm), wooden shapes (flat one of 25.5 x 15 x 0.3 cm; inclined one of 25.5 x 15 x 5.5 cm) or wooden blocks (15 x 10 x 10 cm). A stage of 200 x 70 x 55 cm was placed against the fence within the testing compartment for smaller dogs (pet dogs: Todor, Spike; pack dogs: Bora, Layla, Nia, Zuri) to stand on during the test.

*Experimental trials*

Each cue ended when the two targets on the sides of the table were pushed through the fence for choice-making. When the correct side was chosen, the animal was rewarded. When incorrect, the experimenter shut the screen and set up for the next trial. In case an animal made no choice within roughly 30 seconds after the targets were presented, the trial was ended by pulling the screen down and the cue was repeated. If an animal would not look at the apparatus or the experimenter for 5-10 minutes, would make no choice for 3 consecutive trials or stayed outside the area around the testing table, the session was closed and continued on the next testing day.

*Behavioural coding*

The cue started as soon as the experimenter made eye contact with the animal (look), when the experimenter lifted her hands from underneath the table to start the cue (point, open, reach), as soon as the first cup was lifted (noise) or by the time the screen was completely up (shape, control). In case of the social cues, the cue ended with the experimenter ceasing all actions and looking down while presenting the targets, whereas the causal cues ended after roughly 5 seconds (shape, control) or after the last cup shook and was placed back on the table (noise).

Choice was defined as the first choice made by the animal after the cue had ended and the targets were presented. Choice was coded when the subject either touched one of the two targets with its nose or paw or pushed its nose in or around one of the two food openings. If no direct contact was made or the animal showed no intention to walk over to the targets or to the food openings as soon as the targets were presented, choice was coded when the animal positioned itself in or oriented to the baited or empty area for 3 consecutive seconds (see Supplementary Figure S2 for the different areas). Position overruled orientation, in case an animal would stand at one side, but would stare at the other. If the animal decided to contact one of the targets or food openings within these 3 seconds, this was indicated as its choice.

The relative duration (%) was coded for time that animals positioned themselves in front of the testing table and how long they were out of sight. Additionally, the same division of the apparatus was used to code the orientation of the animals: “orientation to the testing table” and “looking away”. Both position and orientation were coded from the moment the cue started, until the choice was made.

*Data analysis*

First, we compared the number of correct choices in each group (pet dogs, pack dogs and wolves) for the two cue types within each condition. We used generalized linear mixed models with binomial distributions for this purpose. Cue type (look vs. point, open vs. reach, or noise vs. shape), trial (trial 1 or 2) and session (session 1 or 2) were fixed factors. Animal ID was entered as a random factor. These analyses showed that all three groups responded similarly to both cue types in each condition. Accordingly, within each condition, we pooled the data for both cue types for all later analyses.

Groups and the three conditions were then compared for the number of correct choices and the proportion of time spent standing or gazing at the testing table. GLMMs and LMs were used for these analyses, where the fixed factors were animal group (pet dogs, pack dogs, wolves), condition (communicative, behavioural, causal, control), session (session 1 or 2), trial (trial 1 or 2) and cue type (look, point, open, reach, noise, shape, control). Animal ID was entered as a random factor. In the initial models, we checked the three-way interaction between group and condition, and the following two-way interactions: group*condition, group*session, group*trial, group*cue type, session*trial, session*cue type and trial*cue type. Session, trial and cue were entered in all initial models to test for learning effects. However, no significant differences were found either between sessions 1 and 2 or between trials 1 and 2, except for the cue Open, in which a decrease in performance was found in all groups between trial 1 and 2 (GLMM: x^2^ = 6.7808, p < 0.001). Since we found no evidence for an improvement in the animals' performance across trials or sessions, we report the effects of the other factors on all four trials per cue type pooled together.

Additionally, we compared the number of correct choices to chance level for each condition in each group using one-sample t-tests.

*Side bias*

When an animal indicated the same side of the testing table for 13 or 14 trials within a session, it was considered a side bias. Sessions in which this occurred were excluded from the analysis on choice data, but were used for the analyses on position and orientation. A side bias was found in 6 sessions of the pet dogs (2x Cookie, Guinness, Mago, Spike, Tika), 8 sessions of the pack dogs (2x Asali, Bora, 2x Enzi, Maisha, Meru, Sahibu) and 2 sessions of the wolves (2x Kaspar).

**References**

Range, F. & Virányi, Z., 2013. Social learning from humans or conspecifics: differences and similarities between wolves and dogs*. Frontiers in Psycholo*gy, 4(December), pp.1–10. Available at: http://journal.frontiersin.org/article/10.3389/fpsyg.2013.00868/abstract.

**Supplementary Tables**

**Table S1.** List of animals, indicating sex (M, male; MC, castrated male; F, female), age at time of testing, pack size and breed.

| **Individual** | **Group** | **Sex** | **Age (years)** | **Pack size** | **Breed** |
| --- | --- | --- | --- | --- | --- |
| Aramis | Pet dog | M | 3 | - | Giant Schnauzer |
| Cookie | Pet dog | F | 7 | - | Mongrel |
| Guinness | Pet dog | F | 11 | - | Border Collie |
| Hakima | Pet dog | M | 4 | - | Mongrel |
| Hybie | Pet dog | F | 7 | - | Labrador mix |
| Kilio | Pet dog | MC | 5 | - | Mongrel |
| Mago | Pet dog | M | 11 | - | Golden Retriever |
| Michel | Pet dog | MC | 10 | - | Mongrel |
| Spike | Pet dog | MC | 10 | - | Jack Russel x Dachshund |
| Tika | Pet dog | F | 9 | - | Husky x Mongrel |
| Todor | Pet dog | M | 13 | - | Mongrel |
| Tuukka | Pet dog | F | 1 | - | Mongrel |
| Asali | Pack dog | M | 5 | 3 | Mongrel |
| Banzai | Pack dog | M | 1 | 3 | Mongrel |
| Binti | Pack dog | F | 5 | 2 | Mongrel |
| Bora | Pack dog | F | 4 | 3 | Mongrel |
| Enzi | Pack dog | M | 1 | 6 | Mongrel |
| Gombo | Pack dog | M | 1 | 3 | Mongrel |
| Layla | Pack dog | F | 4 | 6 | Mongrel |
| Maisha | Pack dog | M | 5 | 2 | Mongrel |
| Meru | Pack dog | M | 5 | 3 | Mongrel |
| Nia | Pack dog | F | 4 | 3 | Mongrel |
| Nuru | Pack dog | M | 4 | 6 | Mongrel |
| Pepeo | Pack dog | M | 1 | 6 | Mongrel |
| Sahibu | Pack dog | M | 1 | 3 | Mongrel |
| Zuri | Pack dog | F | 4 | 6 | Mongrel |
| Amarok | Wolf | M | 3 | 2 | Timber wolf |
| Aragorn | Wolf | M | 7 | 5 | Timber wolf |
| Chitto | Wolf | M | 3 | 5 | Timber wolf |
| Geronimo | Wolf | M | 6 | 3 | Timber wolf |
| Kaspar | Wolf | M | 7 | 5 | Timber wolf |
| Kenai | Wolf | M | 5 | 2 | Timber wolf |
| Nanuk | Wolf | M | 6 | 2 | Timber wolf |
| Shima | Wolf | F | 7 | 5 | Timber wolf |
| Tala | Wolf | F | 3 | 5 | Timber wolf |
| Una | Wolf | F | 3 | 2 | Timber wolf |
| Wamblee | Wolf | M | 3 | 3 | Timber wolf |
| Yukon | Wolf | F | 6 | 3 | Timber wolf |

**Table S2. List of animals indicating their original place of birth and the litters they belong to. Numbers indicate which individuals are siblings, while letters in combination with a number show the mother of this litter. Stripes mean that that an animal is not related to any of the WSC animals. Com, Behav, Causal and Contr show the % of correct trials within each condition.**

| **Individual** | **Origin** | **Litter** | **Com** | **Behav** | **Causal** | **Contr** |
| --- | --- | --- | --- | --- | --- | --- |
| Kilio | Hungarian shelter | 1 | 62,5 | 50 | 50 | 75 |
| Tuukka | Wolf Science Center | 2 | 75 | 37,5 | 62,5 | 75 |
| Asali | Hungarian shelter | 3 | 50 | 62,5 | 50 | 50 |
| Banzai | Wolf Science Center | 4 | 100 | 50 | 0 | 0 |
| Binti | Hungarian shelter | 3 | 50 | 12,5 | 25 | 75 |
| Bora | Hungarian shelter | 5 | 75 | 50 | 50 | 100 |
| Enzi | Wolf Science Center | 4 | 62,5 | 50 | 62,5 | 25 |
| Gombo | Wolf Science Center | 2 | 50 | 37,5 | 37,5 | 75 |
| Layla | Hungarian shelter | A4, 5 | 87,5 | 62,5 | 50 | 75 |
| Maisha | Hungarian shelter | 1 | 50 | 25 | 75 | 0 |
| Meru | Hungarian shelter | - |  |  |  |  |
| Nia | Hungarian shelter | B2 | 50 | 87,5 | 50 | 25 |
| Nuru | Hungarian shelter | 6 | 62,5 | 75 | 25 | 50 |
| Pepeo | Wolf Science Center | 4 | 62,5 | 37,5 | 37,5 | 75 |
| Sahibu | Wolf Science Center | 2 | 75 | 50 | 50 | 50 |
| Zuri | Hungarian shelter | 6 | 75 | 37,5 | 37,5 | 50 |
| Amarok | Minnesota Wildlife Connection, USA | 7 | 62,5 | 62,5 | 75 | 75 |
| Aragorn | Tierwelt Herberstein, Austria | 8 | 75 | 62,5 | 50 | 75 |
| Chitto | Minnesota Wildlife Connection, USA | 9 | 62,5 | 50 | 87,5 | 50 |
| Geronimo | Triple D Farm, Montana, USA | 10 | 75 | 50 | 50 | 75 |
| Kaspar | Tierwelt Herberstein, Austria | - |  |  |  |  |
| Kenai | Parc Safari, Canada | - |  |  |  |  |
| Nanuk | Triple D Farm, Montana, USA | - |  |  |  |  |
| Shima | Tierwelt Herberstein, Austria | 8 | 87,5 | 37,5 | 75 | 25 |
| Tala | Minnesota Wildlife Connection, USA | 7 | 87,5 | 37,5 | 87,5 | 75 |
| Una | Minnesota Wildlife Connection, USA | 9 | 62,5 | 37,5 | 37,5 | 0 |
| Wamblee | Haliburton Forest, Canada | - |  |  |  |  |
| Yukon | Triple D Farm, Montana, USA | 10 | 87,5 | 62,5 | 62,5 | 0 |

**Table S3.** All the statistical values linked to the choice data.

| **Model terms** | **Estimate** | **SE** | **z or t** | **Pr(>|z|) or Pr(>|t|)** | ***df*** | **Chisq** | **Pr**  **(>Chisq)** |
| --- | --- | --- | --- | --- | --- | --- | --- |
| *Cues within conditions* |  |  |  |  |  |  |  |
| *Look vs Point* |  |  |  |  |  |  |  |
| Pet dogs |  |  |  |  | 1 | 0.068 | 0.795 |
| Pack dogs |  |  |  |  | 1 | 0.234 | 0.629 |
| Wolves |  |  |  |  | 1 | 0.910 | 0.340 |
| *Open vs Reach* |  |  |  |  |  |  |  |
| Pet dogs |  |  |  |  | 1 | 0.251 | 0.617 |
| Pack dogs |  |  |  |  | 1 | 0.056 | 0.814 |
| Wolves |  |  |  |  | 1 | 0.182 | 0.670 |
| *Noise vs Shape* |  |  |  |  |  |  |  |
| Pet dogs |  |  |  |  | 1 | 0.999 | 0.318 |
| Pack dogs |  |  |  |  | 1 | 0.000 | 1.000 |
| Wolves |  |  |  |  | 1 | 1.283 | 0.257 |
|  |  |  |  |  |  |  |  |
| Group |  |  |  |  | 2 | 5.746 | 0.057 |
| Pet dogs vs Pack dogs | 0.014 | 0.182 | 0.078 | 0.938 |  |  |  |
| Pack dogs vs Wolves | 0.360 | 0.169 | 2.127 | **0.033** |  |  |  |
| Condition |  |  |  |  | 3 | 21.077 | **<0.001** |
| Group*Condition |  |  |  |  | 6 | 9.393 | 0.153 |
|  |  |  |  |  |  |  |  |
| *Communicative cues* |  |  |  |  |  |  |  |
| Group |  |  |  |  | 2 | 1.523 | 0.467 |
| Pet dogs vs Pack dogs | -0.076 | 0.355 | -0.213 | 0.831 |  |  |  |
| Pack dogs vs Wolves | 0.327 | 0.340 | 0.961 | 0.337 |  |  |  |
| Session |  |  |  |  | 1 | 0.040 | 0.842 |
| Trial |  |  |  |  | 1 | 0.503 | 0.478 |
| Group*Session |  |  |  |  | 2 | 3.980 | 0.137 |
| Group*Trial |  |  |  |  | 2 | 0.712 | 0.700 |
| Session*Trial |  |  |  |  | 1 | 0.432 | 0.511 |
| Above chance pet dogs |  |  | 2.882 | **0.018** | 9 |  |  |
| Above chance pack dogs |  |  | 3.742 | **0.003** | 11 |  |  |
| Above chance wolves |  |  | 4.822 | **0.001** | 10 |  |  |
|  |  |  |  |  |  |  |  |
| *Behavioural cues* |  |  |  |  |  |  |  |
| Group |  |  |  |  | 2 | 0.046 | 0.977 |
| Pet dogs vs Pack dogs | -0.073 | 0.340 | -0.213 | 0.831 |  |  |  |
| Pack dogs vs Wolves | -0.038 | 0.313 | -0.122 | 0.903 |  |  |  |
| Session |  |  |  |  | 1 | 3.735 | 0.053 |
| Trial |  |  |  |  | 1 | 0.439 | 0.508 |
| Group*Session |  |  |  |  | 2 | 1.707 | 0.426 |
| Group*Trial |  |  |  |  | 2 | 2.603 | 0.272 |
| Session*Trial |  |  |  |  | 1 | 0.524 | 0.469 |
| Above chance pet dogs |  |  | -0.580 | 0.576 | 9 |  |  |
| Above chance pack dogs |  |  | -0.352 | 0.732 | 11 |  |  |
| Above chance wolves |  |  | -0.690 | 0.506 | 10 |  |  |
|  |  |  |  |  |  |  |  |
| *Causal cues* |  |  |  |  |  |  |  |
| Group |  |  |  |  | 2 | 13.069 | **0.001** |
| Pet dogs vs Pack dogs | 0.302 | 0.343 | 0.881 | 0.379 |  |  |  |
| Pack dogs vs Wolves | 1.138 | 0.326 | 3.486 | **<0.001** |  |  |  |
| Session |  |  |  |  | 1 | 0.121 | 0.728 |
| Trial |  |  |  |  | 1 | 0.018 | 0.893 |
| Group*Session |  |  |  |  | 2 | 2.297 | 0.317 |
| Group*Trial |  |  |  |  | 2 | 3.706 | 0.157 |
| Session*Trial |  |  |  |  | 1 | 0.015 | 0.902 |
| Above chance pet dogs |  |  | -1.137 | 0.285 | 9 |  |  |
| Above chance pack dogs |  |  | -1.750 | 0.108 | 11 |  |  |
| Above chance wolves |  |  | 3.155 | **0.010** | 10 |  |  |
|  |  |  |  |  |  |  |  |
| *Control* |  |  |  |  |  |  |  |
| Group |  |  |  |  | 2 | 0.193 | 0.908 |
| Pet dogs vs Pack dogs | -0.206 | 0.563 | -0.365 | 0.715 |  |  |  |
| Pack dogs vs Wolves | -0.205 | 0.524 | -0.392 | 0.695 |  |  |  |
| Session |  |  |  |  | 1 | 0.117 | 0.732 |
| Trial |  |  |  |  | 1 | 0.039 | 0.845 |
| Group*Session |  |  |  |  | 2 | 0.475 | 0.788 |
| Group*Trial |  |  |  |  | 2 | 3.074 | 0.215 |
| Session*Trial |  |  |  |  | 1 | 0.032 | 0.857 |
| Above chance pet dogs |  |  | 0.000 | 1.000 | 9 |  |  |
| Above chance pack dogs |  |  | 0.233 | 0.820 | 11 |  |  |
| Above chance wolves |  |  | 0.000 | 1.000 | 10 |  |  |

**Table S4.** All the statistical values linked to the position data.

| **Model terms** | **Estimate** | **SE** | **t** | **Pr(>|t|)** | ***df*** | **F** | **Pr(>F)** |
| --- | --- | --- | --- | --- | --- | --- | --- |
| Group |  |  |  |  |  | 12.805 | **<0.001** |
| Pet dogs vs Pack dogs | 0.016 | 0.045 | 0.356 | 0.724 | 35.100 |  |  |
| Pack dogs vs Wolves | -0.192 | 0.045 | -4.305 | **<0.001** | 35.100 |  |  |
| Condition |  |  |  |  |  | 13.036 | **<0.001** |
| Group*Condition |  |  |  |  |  | 2.158 | **0.045** |

**Table S5.** All the statistical values linked to the orientation data.

| **Model terms** | **Estimate** | **SE** | **t** | **Pr(>|t|)** | ***df*** | **F** | **Pr(>F)** |
| --- | --- | --- | --- | --- | --- | --- | --- |
| Group |  |  |  |  |  | 9.486 | **<0.001** |
| Pet dogs vs Pack dogs | 0.053 | 0.058 | 0.912 | 0.368 | 35.100 |  |  |
| Pack dogs vs Wolves | -0.193 | 0.058 | -3.353 | **0.002** | 35.100 |  |  |
| Condition |  |  |  |  |  | 22.281 | **<0.001** |
| Group*Condition |  |  |  |  |  | 1.832 | 0.090 |

**Table S6.** The statistical values from a Tukey’s HSD test for pet dog orientation data to compare cue conditions.

|  | Communicative cues | Behavioural cues | Causal cues |
| --- | --- | --- | --- |
| Behavioural cues | 0.999 | **-** | - |
| Causal cues | **<0.001** | **<0.001** | - |
| Control cue | 0.972 | 0.988 | **0.004** |

**Table S7.** The statistical values from a Tukey’s HSD test for pack dog orientation data to compare cue conditions.

|  | Communicative cues | Behavioural cues | Causal cues |
| --- | --- | --- | --- |
| Behavioural cues | 0.437 | **-** | - |
| Causal cues | **0.016** | **<0.001** | - |
| Control cue | 0.359 | **0.023** | 0.853 |

**Table S8.** The statistical values from a Tukey’s HSD test for wolf orientation data to compare cue conditions.

|  | Communicative cues | Behavioural cues | Causal cues |
| --- | --- | --- | --- |
| Behavioural cues | 0.916 | **-** | - |
| Causal cues | 0.061 | 0.249 | - |
| Control cue | 0.130 | 0.349 | 0.999 |


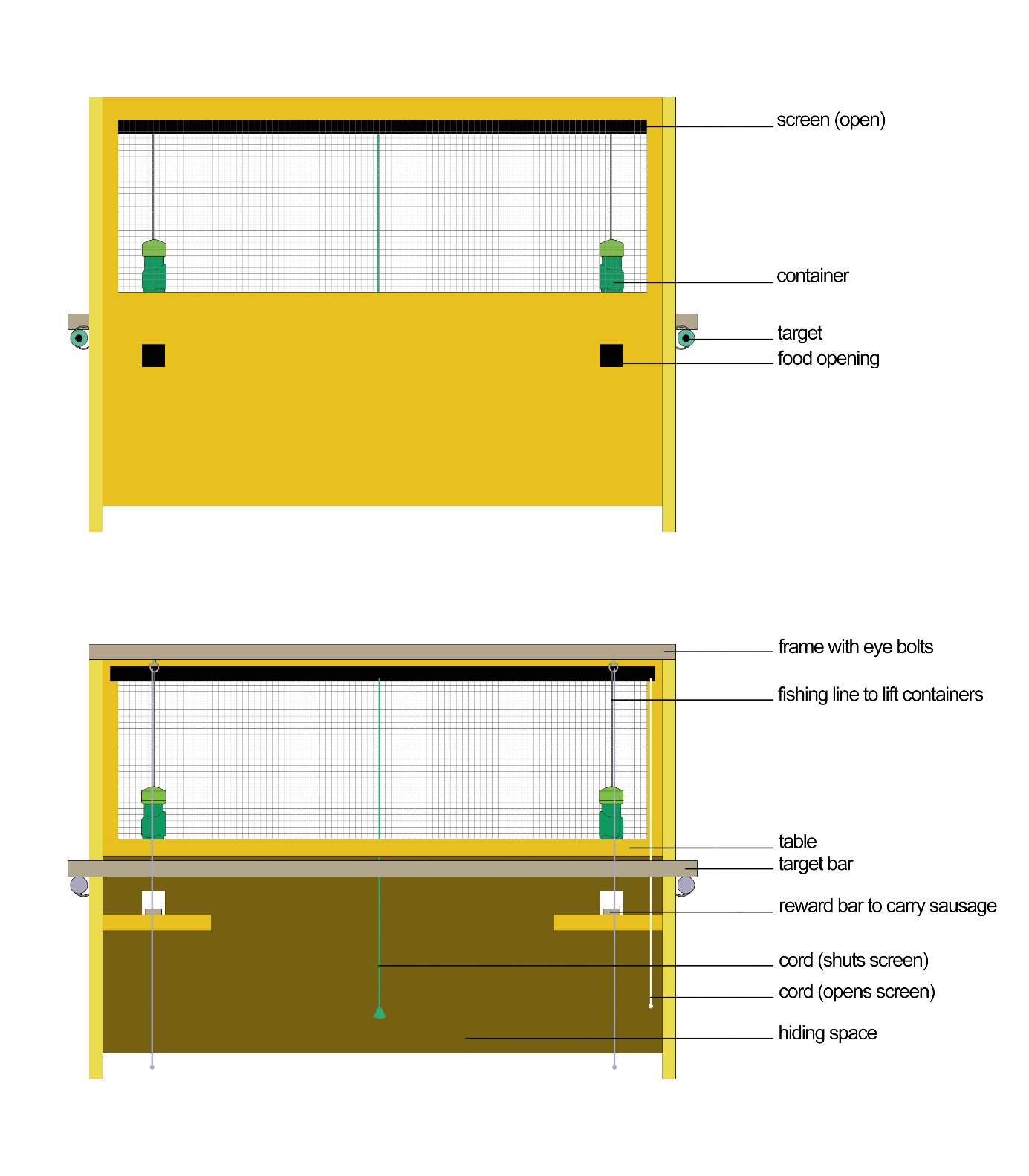
**Supplementary Figures**

**Figure S1.** The experimental set-up of the apparatus from the animal’s perspective (top drawing) and from the experimenter’s perspective (bottom drawing).


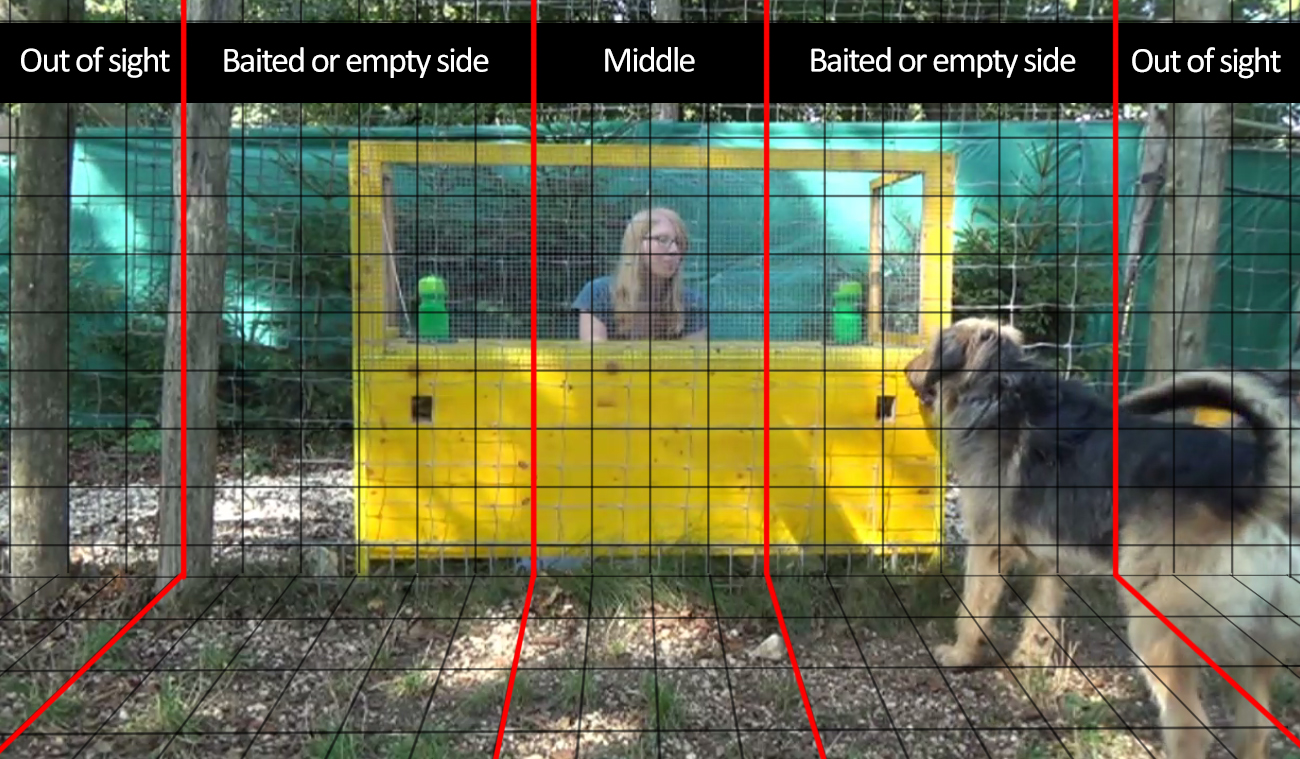
**Figure S2.** The different areas that were used for coding choice (baited, empty) and for the position and orientation (the area of baited, middle and empty side taken together as the testing table) of the animals during each cue.


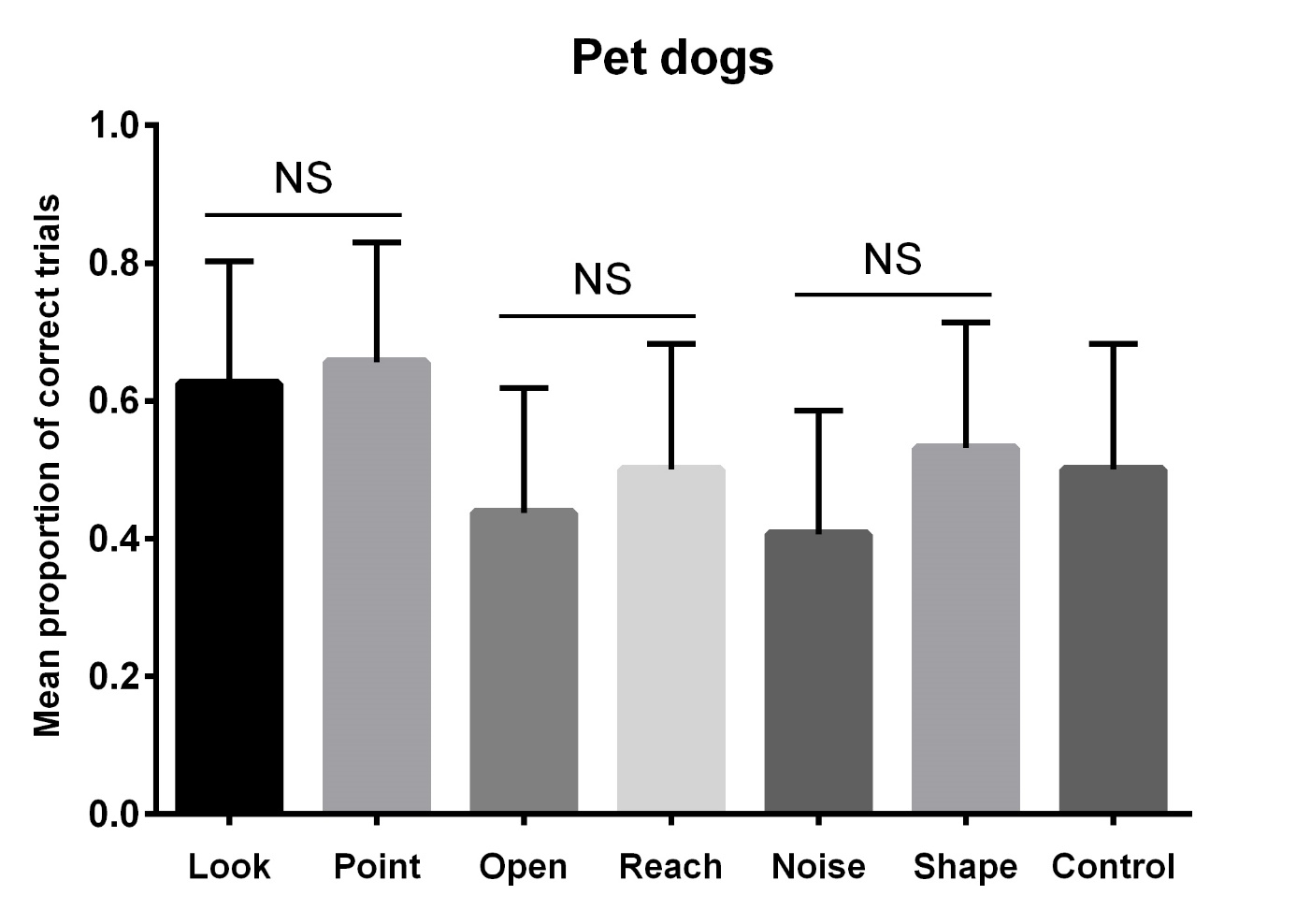
**Figure S3.** The mean proportion of correct trials (including 95% confidence interval) for the pet dog group per cue.


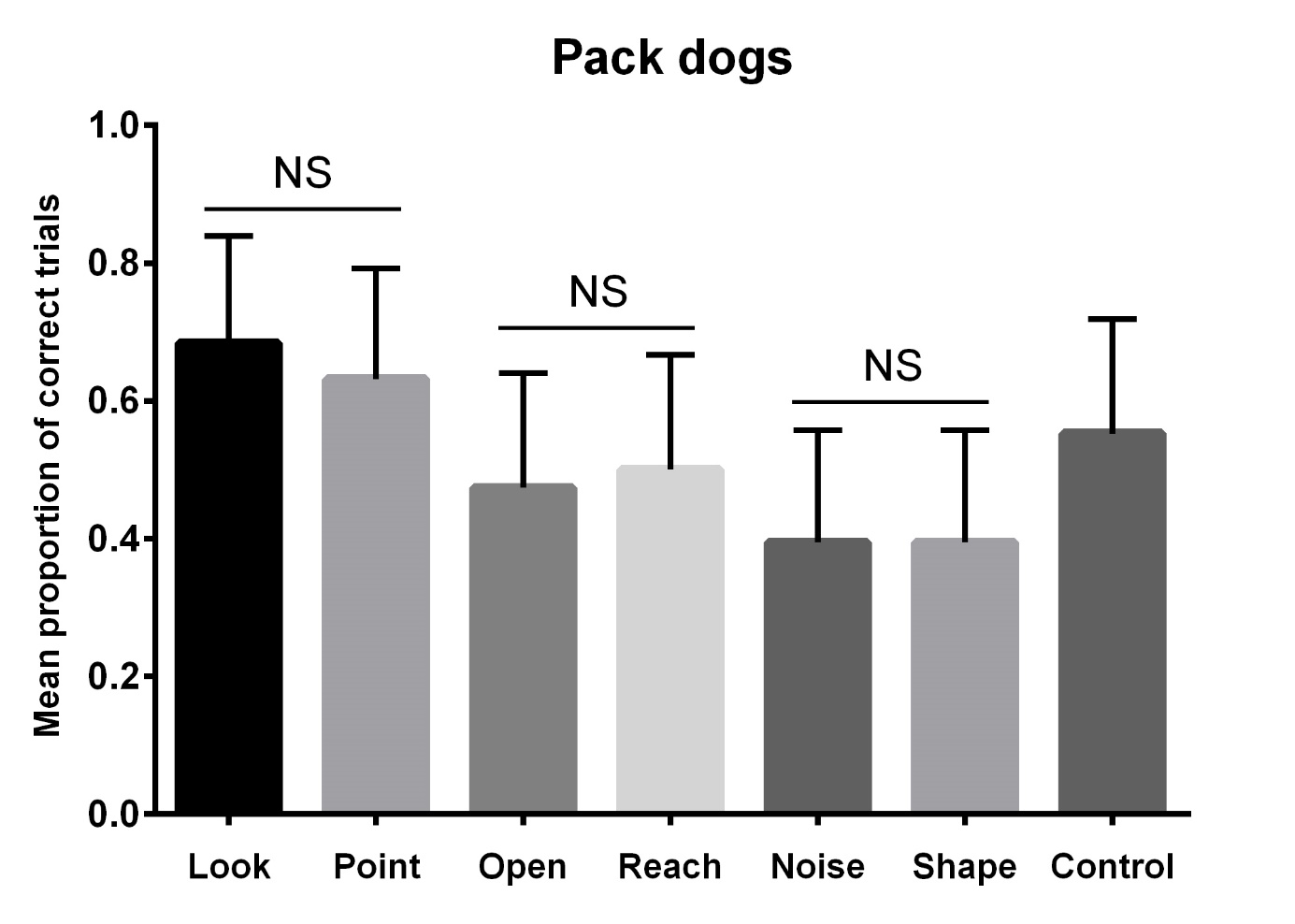
**Figure S4.** The mean proportion of correct trials (including 95% confidence interval) for the pack dog group per cue.


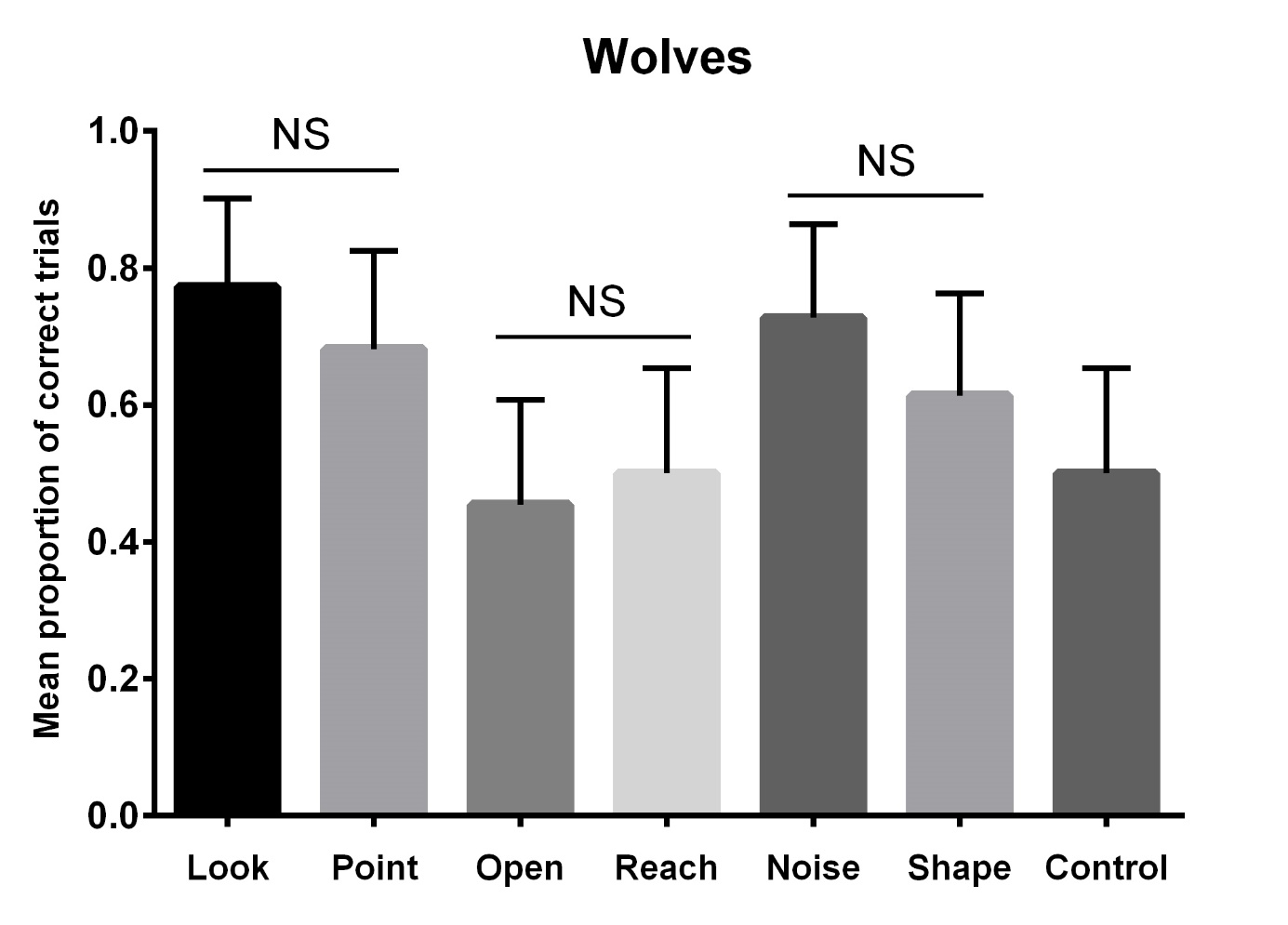
**Figure S5.** The mean proportion of correct trials (including 95% confidence interval) for the wolf group per cue.


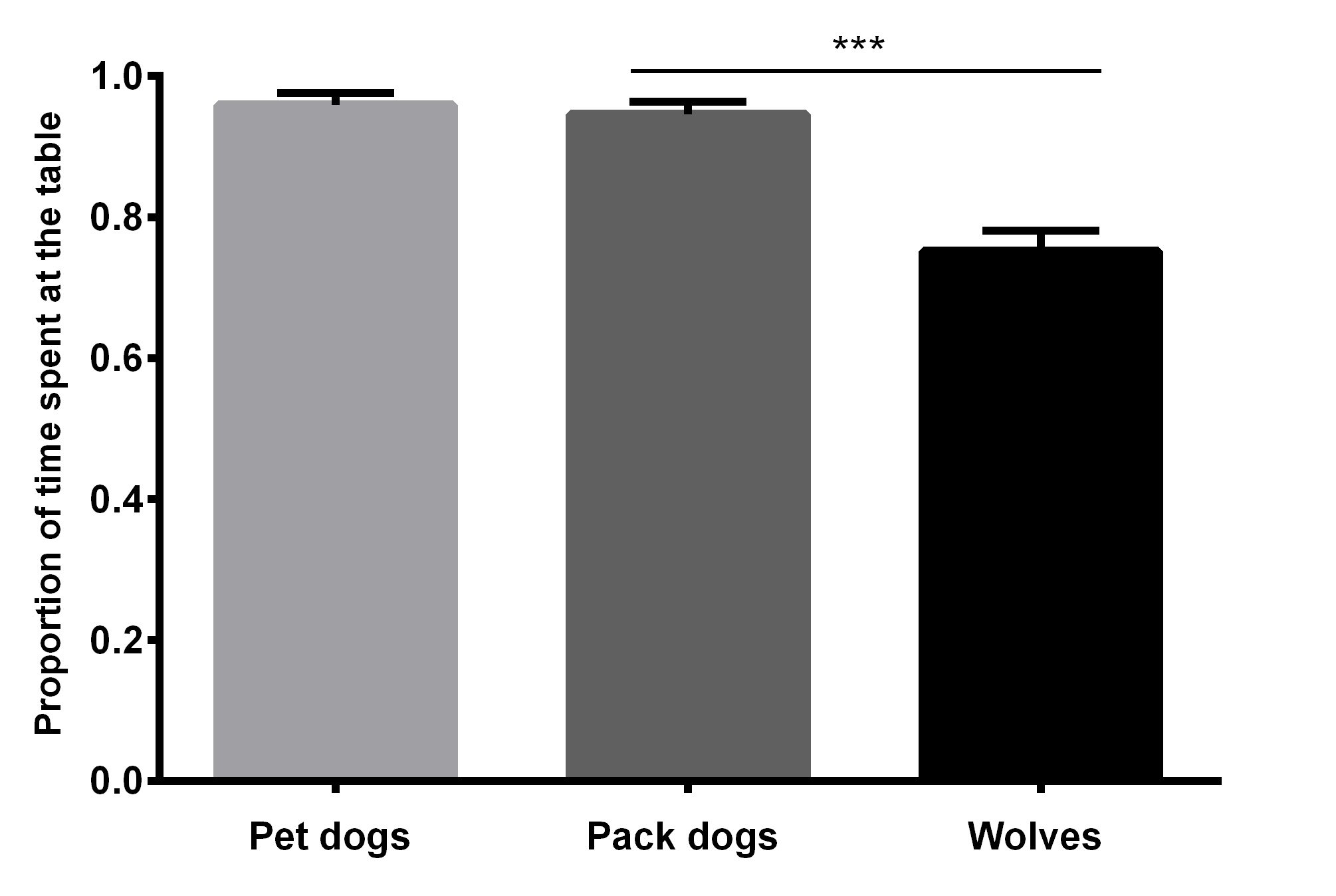
 **Figure S6.** Mean proportion of time (including 95% confidence interval) that the groups were positioned in front of the testing table. Significant difference is indicated by asterisk (* p<0.05, ** p<0.01, *** p<0.001).


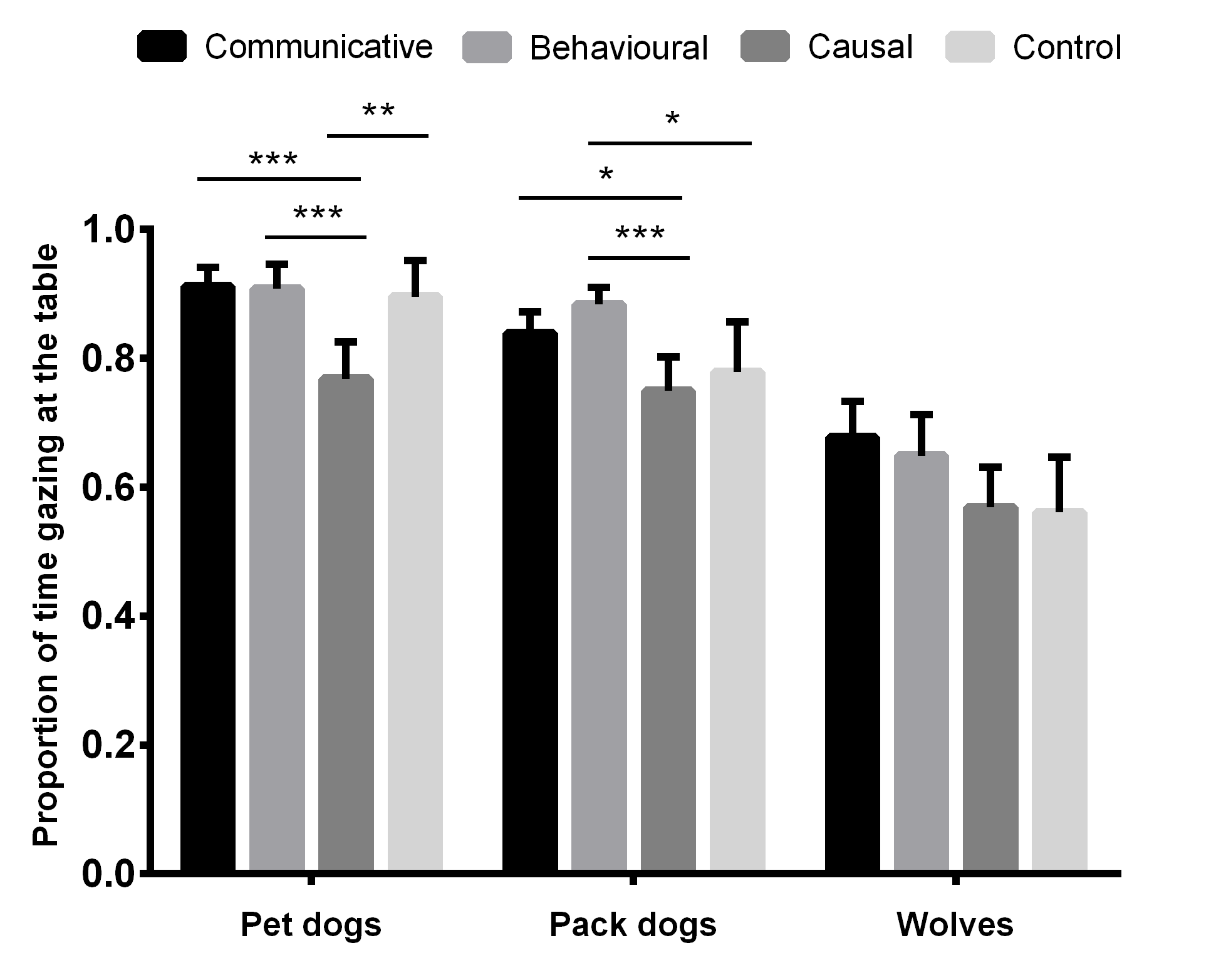
**Figure S7.** Mean proportion of time (including 95% confidence interval) that the groups gazed at the testing table for the four different cue conditions. Significant differences are indicated by asterisk (* p<0.05, ** p<0.01, *** p<0.001).
